# Herpes in Pregnancy

**DOI:** 10.1155/S1064744994000293

**Published:** 1994

**Authors:** Curtis R. Cook, Stanley A. Gall

**Affiliations:** Department of Obstetrics and Gynecology University of Louisville School of Medicine Louisville KY 40292 USA

## Abstract

The management of genital herpesvirus infections in pregnancy has seen many changes over the past decade as we have continued to learn more about the epidemiology of the disease. This article reviews these changes and highlights ongoing controversies. Clinical management schemes are proposed based upon this most recent information.


Infectious Diseases in Obstetrics and Gynecology 1:298–304 (1994)
© 1994 Wiley-Liss, Inc.

### Herpes in Pregnancy

**Curtis R. Cook and Stanley A. Gall**
*Department of Obstetrics and Gynecology, University of Louisville School of Medicine, Louisville, KY*

ABSTRACT

**The management of genital herpesvirus infections in pregnancy has seen many changes over the past decade as we have continued to learn more about the epidemiology of the disease. This article reviews these changes and highlights ongoing controversies. Clinical management schemes are proposed based upon this most recent information.** © 1994 Wiley-Liss, Inc.

KEY WORDS
Herpes simplex virus, genital herpes, neonatal herpes

The management of genital herpesvirus infections in pregnancy continues to be a challenge in the 1990s. Although much has been learned about the natural history and pathophysiology of this disease, many questions remain unanswered. The purpose of this article is to review recent developments as well outline areas of controversy where further investigation is needed. Finally, a management scheme will be proposed incorporating our best information to date on this challenging issue.

The estimated incidence of neonatal infection from herpes simplex virus (HSV) ranges from 1/7,500 to 1/30,000 livebirths,[1] with evidence of increasing frequency as high as 1/2,000 to 1/3,500 livebirths over the past 2 decades.[2,3] These are frequently life-threatening infections with more than 40% of neonates dying or suffering from significant neurologic sequelae despite the use of antiviral treatments.[4,5] Unfortunately, most cases of neonatal infection are not currently preventable, with 85% of HSV-infected infants born to asymptomatic women.[4,6] The challenge for the future lies in identifying these asymptomatic infectious women in a reliable, timely, and cost-effective manner in order to prevent a greater number of neonatal infections.

### NATURAL HISTORY

HSVs are double-stranded DNA viruses categorized into 2 types based upon their immunologic and clinical differences. Type 1 (HSV-1) most commonly causes nongenital herpes infections, but may be responsible for approximately 15% of genital infections.[7,8] Type 2 (HSV-2) affects predominantely the genital regions, but may also occasionally cause orolabial lesions. Genital HSV-1 infections present less concern for maternal or neonatal complications. Unfortunately, there is no reliable method for differentiating these infections clinically or by commercially available laboratory studies. It has also been noted that only 15–50% of HSV-1 compared with 70–90% of HSV-2 genital infections will recur, with more than 90% of the recurrences being secondary to HSV-2.[8–10]

Recently, the advent of type-specific glycoprotein assays has allowed researchers to determine the prevalence of seropositivity for HSV-2. This number varies by population, approaching 100% in prostitutes. The rate of seropositivity in various cohorts of patients in the United States is approximately 20–40%.[11,12] As many as 80% of these patients do not recall a primary infection and represent asymptomatic primary infections.[12,13] Presum-

**Address correspondence/reprint requests to Dr. Curtis R. Cook, Department of Obstetrics and Gynecology, University of Louisville, Louisville, KY 40292.**

Received February 18, 1994

Accepted April 22, 1994

Review Article ably, some of these patients would have been infected in a less symptomatic region such as the cervix or upper vagina. Others probably had ameliorated primary infections secondary to the presence of protective antibodies from a prior nongenital herpes infection. Prospective studies have shown an annual conversion rate to HSV-2 of 0.6% in previously seronegative women.[14]

HSV replication and dissemination occur by direct cell-to-cell transmission. The presence of HSV antibodies does not prevent recurrences, but may limit dissemination and viremia. The virus is known to remain dormant in the dorsal root ganglia from where it may reappear as asymptomatic shedding or symptomatic clinical disease. It is not clear what triggers these recurrences although correlation has been noted with fever, trauma, stress, menstruation, and immunosuppression. Pregnancy is not felt to exacerbate the disease.

Clinically, HSV infections are categorized as 1st-episode primary, 1st-episode nonprimary, or recurrent. Primary infections occur without the presence of previous antibodies to either HSV-1 or HSV-2. First-episode nonprimary infections occur if either antibody is already present, providing some protection and blunting of symptoms. Finally, recurrent infections occur in individuals with a history of genital herpes.

Primary HSV infections provide the greatest concern, particularly when they occur near the time of delivery. Infants born to these mothers are believed to be at increased risk because of the lack of protective maternal antibodies, the longer duration of shedding larger viral loads, and the increased rate of cervical involvement. More than 80% of primary infections have associated cervical shedding at delivery,[9] with a vertical transmission rate of 40–80%.[15]

Primary genital herpes infections are marked by a 3–6 day incubation period after exposure. Vulvar burning and pruritis usually precede the presence of multiple vesicles that may involve the vulva, vagina, and cervix. These lesions become shallow, painful ulcers after 24–36 h before eventually crusting over. There are usually in excess of 20 vesicles that contain between 0.5–1 million viral particles/vesicle.[16] Lesions persist for an average of 19.7 days with viral shedding for 11.8 days on average. More than 2/3 of these individuals will have constitutional flu-like symptoms which may include fever and painful lymphadenopathy.[9] Antibodies to HSV are noted about 7 days following the onset of primary infection and peak in 2–3 weeks. Infected patients then remain seropositive for life.

Rarely, individuals may develop disseminated disease including meningitis, hepatitis, encephalitis, and pneumonitis. Herpes meningitis has been noted in approximately 4–8% of primary infections with other visceral involvement being much less commonly noted.[17] Overall, these can be quite serious infections with maternal and neonatal mortalities in excess of 50%. Recently, the use of systemic antiviral agents such as acyclovir has resulted in improved maternal and neonatal outcomes for these severe cases.[18]

Primary infections have been associated with an increased rate of preterm delivery.[15] Indeed, 50% of HSV-infected infants are born premature. Of the women with primary infections, 80% will be expected to have 2–4 recurrences during pregnancy.[19–21] Many of these recurrences will be asymptomatic, which underscores the current clinical dilemma.

First-episode nonprimary infections are less symptomatic due to the protective effect of HSV antibodies. These infections occur less commonly than primary infections. On average, these lesions persist for 15.5 days and continue to shed virus for 6.8 days. Only 16% of these cases are expected to demonstrate systemic symptoms.[9]

Recurrent HSV infections comprise the great majority of infections occurring at the time of delivery. The management of these patients remains the most controversial, as is noted later in this article. Recurrent infections are associated with viral shedding only 12–15% of the time.[9,20,21] The vertical transmission rate is estimated at less than 5%.[1,22,23] This is apparently secondary to the protection of maternally acquired antibodies and the presence of a decreased viral inoculum. Recurrent infections have not been associated with an increased risk for preterm labor.[19–21] Lesions persist for an average of 9.3 days with viral shedding for an average of 3.9 days. These lesions are fewer in number and generally less painful. Systemic symptoms occur in less than 10% and disseminated cases are extremely rare.[9] Recurrences are noted more frequently late in pregnancy, but unfortunately have no predictable correlation to viral shedding.[24–26] Antibody titers do not rise significantly with recurrences and are therefore not helpful in confirming these events.

### DIAGNOSIS

The "gold standard" for diagnosis of HSV infections is still isolation by viral culture. Most cultures will be positive by 48–72 h with a sensitivity of 90–95%, although 7–10 days are necessary to confirm a negative result. Even culture becomes less than 50% sensitive if the lesions are already crusted over.[27] For this reason, it is preferable to culture lesions early in the vesicular or ulcerative stage. Other tests have been developed including monoclonal antibody tests and enzyme-linked immunosorbent assays (ELISA). These alternative tests perform best in high-prevalance populations, but still lack the sensitivity and specificity of viral culture. Cytologic evaluation using the Papanicolaou smear or the Tzanck preparation for the presence of intranuclear inclusions or multinucleated giant cells has also been used for rapid evaluation. This is helpful when positive, but is limited by its relatively low sensitivity and high false negative rate. Recently, investigators have used polymerase chain reaction (PCR) to isolate viral fragments.[28] The ability to distinguish type-2 glycoprotein G has allowed virotyping for research purposes.[11–13] However, no reliable commercial assay is available.[29]

### NEONATAL OUTCOMES

Neonatal HSV infections can be life-threatening events with significant ocular and neurologic sequelae. These sequelae include microophthalmia, retinal dysplasia, chorioretinitis, microcephaly, mental retardation, seizure, apnea, and coma. Infections usually arise from direct contact with the virus during passage through the birth canal or from ascending infections after rupture of the membranes. Approximately 10% of cases arise postnatally from direct contact with parents or caretakers after delivery.[4] Less commonly, infections may occur as congenital infections after transplacental passage of the virus.[30,31]

Neonatal infections may be disseminated, localized, or asymptomatic. Disseminated illness more frequently follows primary infections and may have a 50–60% mortality with serious neurologic sequelae in 50% of survivors.[5] Infections with HSV-2 appear to be associated with a worse prognosis.[32] The prognosis also appears to be worsened by delay in the initiation of antiviral agents.[4] Localized infections usually involve the eyes, skin, or mucous membranes and tend to do well, although long-term morbidities may result. Some infants remain asymptomatic throughout the neonatal period. Higher levels of neutralizing antibodies have been demonstrated in the amniotic fluid and cord bloods of the less symptomatic infants.[33]

Intrauterine transplacental infections are responsible for approximately 5% of the infections and carry a mortality rate of 30%.[30] These infections are marked by early symptoms of skin scarring, rash, microophthalmia, chorioretinitis, microcephaly, and intrauterine growth restriction. Amniocentesis has not been effective in predicting these infections. Because of the rarity of these infections, therapeutic abortions are not recommended for primary genital herpes infections occurring in the 1st trimester.

Overall, infected infants are expected to demonstrate symptoms by 1–2 weeks of life with some cases not presenting until 4–6 weeks of age. Typical symptoms are nonspecific and include seizure, lethargy, irritability, temperature instability, poor feeding, and skin manifestations. Antiviral agents have been effective if initiated early. One recent analysis calculated an overall mortality of 18.3% with severe sequelae in 15.4% and moderate sequelae in 10.1% with recovery in 56.2% of infected infants.[34] The Collaborative Antiviral Study Group reported morbidities and mortalities of 17% and 60% in disseminated disease, 67% and 14% in isolated central nervous system (CNS) disease, and 8% and 0% in disease localized to the portal of entry.[35]

### CLINICAL MANAGEMENT

Until the mid-1980s, the usual practice to identify infectious women in labor was to perform weekly herpes cultures of the lower genital tract in those women with a history of genital herpes. Cesarean delivery was then recommended for those women with positive cultures near the time of partuition or with lesions present in labor. Such surveillance cultures were subsequently shown to be ineffective in predicting the presence of virus at delivery in asymptomatic women.[22,26] In addition, these cultures were estimated to cost $1.8 million/case of neonatal herpes averted.[36] For these reasons, the

Infectious Disease Society for Obstetrics and Gynecology recommended the abandonment of surveillance cultures as well as amniocentesis to predict the presence of virus at delivery. Cesarean delivery was still recommended for the presence of lesions or prodromal symptoms in labor.[37] The limitations of this recommendation were recognized including the inability to prevent transplacental infections and to detect asymptomatic cervical shedding.

Obstetricians are still frustrated by the inability to prevent more cases of neonatal herpes through the use of cesarean deliveries. Currently, 20–30% of neonatal infections occur in infants delivered by cesarean section.[4,6] Some of these may have occurred as a result of ascending infections after rupture of the membranes. However, 8% of neonatal infections occur despite cesarean delivery with intact membranes.[6] The biggest challenge still remains in identifying the 85% of neonatal infections resulting from asymptomatic shedding. We are still without a sensitive, timely, and inexpensive screening test to apply to the general pregnant population in order to identify these high-risk women.

With these limitations, the most prudent approach emphasizes identifying those women with the highest risk for cervical shedding at delivery in order to minimize contact between the fetus and the virus. This approach would include eliciting a history of genital herpes from either partner and confirming new lesions with HSV cultures if not previously documented. Surveillance cultures should be abandoned unless used to demonstrate the absence of virus from a late-trimester primary infection. Patients should be asked to present early after rupture of the membranes and should be examined carefully for the presence of external genital lesions while in labor. Visual inspection has not proved beneficial for detecting cervical or vaginal lesions. Cesarean delivery is still recommended if lesions are noted in labor regardless of the length of membrane rupture. In the absence of documented lesions or prodrome, vaginal delivery may be attempted regardless of a history of genital herpes. Procedures that may allow additional portals of entry such as scalp electrodes and scalp sampling should be avoided, if possible, but are not contraindicated in the absence of lesions.

Neonates should be initially isolated, cultured, observed, and possibly treated with acyclovir if a sufficient index of suspicion exists. Mothers need not be isolated from their infant or other patients. Mothers should practice thorough handwashing and avoid infant contact with lesions. Breastfeeding may be allowed as long as there are no lesions on the breast. Prolonged in-hospital observation is not felt necessary in the absence of a positive neonatal cultue or unusual symptoms.. These mothers need to be educated about presenting neonatal symptoms and need to report immediately any skin lesions or mental status changes. Mothers with a history of genital herpes should be aware of the small risk for neonatal infection even if they are asymptomatic at delivery.

### CURRENT CONTROVERSIES

There still remain several controversial areas within the management of genital herpes in pregnancy. The 1st involves the use of antiviral agents. Acyclovir, a specific viral DNA polymerase inhibitor, has been demonstrated to reduce the duration of symptoms in nonpregnant primary infections. It has also been shown to reduce the severity and number of recurrences in nonpregnant patients. Its use is not currently recommended in pregnancy unless disseminated disease exists. However, acyclovir has been used in hundreds of pregnant women without adverse fetal outcomes.[38,39] Its use has been proposed to attempt reduction in herpes recurrences and subsequent cesarean delivery for this indication. Preliminary data have been promising in this regard;[40,41] however, this therapy may still not prevent asymptomatic viral shedding and potential neonatal infection.[42] Furthermore, the use of acyclovir may delay the humoral response to HSV, thereby preventing or reducing passive immunization of the fetus.[43] At this time, acyclovir cannot be recommended except in research protocols and in cases of life-threatening disseminated illness. Of course, acyclovir will continue to be used in neonates and nonpregnant patients.

Second, the management of premature rupture of the membranes (PROM) in the presence of HSV lesions also remains an area of controversy. Case reports and case series have demonstrated the possibility of expectant management in preterm PROM.[44,45] In the largest reported series of 18 patients, 3 patients suffered additional recurrences during their latency period. Eight patients were delivered by cesarean section for the presence of lesions at the time of indication for delivery. None of the 18 infants developed neonatal HSV infections, although some of the mothers were treated with acyclovir antepartum.[45] Based on these limited data, it would seem reasonable to perform cesarean deliveries if at or near term and otherwise to manage the remote-from-term patient expectantly unless delivery is indicated. There are still insufficient data to recommend the antepartum use of acyclovir.

Previously, it had been recommended to perform a cesarean delivery within 4–6 h of rupture of the membranes at term. A vaginal delivery was allowed if ruptured beyond this period of time. This recommendation was based on 2 small patient series that demonstrated no infections if ruptured for fewer than 4 h with significant infections despite cesarean delivery if ruptured beyond this point.[46,47] Unfortunately, subsequent data have demonstrated infections even if the membranes were intact or ruptured fewer than 4 h. Additionally, prolonged latency periods do not ensure neonatal infection.[44,45] Therefore, it still seems prudent to perform cesarean deliveries in the presence of lesions in labor regardless of the length of membrane rupture.

Third, another area of controversy involves the intrapartum management of lesions away from the birth canal. Lesions involving distal locations such as the thigh and buttock have been associated with neonatal infections. However, the risk of concurrent cervical shedding is very low.[21,25] For this reason, it would seem reasonable to allow vaginal delivery with covering of distal lesions to prevent neonatal contact.

Fourth, the recommendation of the Infectious Disease Society for Obstetrics and Gynecology to obtain maternal cultures at delivery in asymptomatic women with a history of genital herpes remains controversial.[37] This was initially proposed in order to guide further neonatal therapy. Unfortunately, cultures are less sensitive in this group of women because of the decreased viral load. Additionally, few infants exposed to this clinical situation will develop neonatal infection. This brings into question the cost-benefit of this approach. It would seem more reasonable to culture women with lesions at delivery and neonates at increased risk for exposure to cervical shedding.

Finally, the most recent area of controversy involves the delivery management of patients with recurrent rather than primary infections. These infections make up the vast majority of genital infections occurring at the time of delivery. There is good evidence to support the premise that we have overestimated the risk of cervical shedding and subsequent neonatal infection in the patients with recurrent infections.[22] Additionally, it has been recently estimated that our current management of these patients would cost $2.5 million/case of neonatal infection averted.[34] In this same decision analysis, an estimated 1,580 cesarean sections and 0.57 maternal deaths would occur to prevent each case of neonatal infection in women with recurrent genital herpes. In contrast, only 9 cesarean sections and 0.004 maternal deaths would occur in cases of women with primary genital herpes infections. This would result in an overall cost savings of $38,000/case of neonatal infection averted in these primary infections.[34] While this study is provocative, there are some limitations of concern in the analysis. Several of the assumptions made were classified as "low level of confidence" or "best guess" because of a paucity of data. This information should, however, generate further discussion and evaluation of our management of this group of patients with recurrent genital herpes infections. Furthermore, it now seems justifiable to pursue randomized trials of delivery management for patients with recurrent infections if patients and practitioners can be convinced to participate.

### CONCLUSIONS

We have learned much about the natural history and pathophysiology of genital herpes infections in pregnancy over the past 2 decades. However, many questions remain unanswered. How do we effectively screen for asymptomatic cervical shedding in a reliable and cost-effective manner? How do we best manage the patient with a history of genital herpes and recurrent lesions in labor? What, if any, therapies are effective in reducing vertical transmission? These and other questions will continue to be areas of active research. Until such time that answers to these and other questions are available, we will continue to focus on identifying the patient at greatest risk for cervical shedding in labor and attempt to minimize neonatal contact with such shedding.

### REFERENCES

1. Chuang T-Y: Neonatal herpes: Incidence, prevention and consequences. Am J Prev Med 4:47–53, 1988.
2. Sullivan-Bolyai J, Hull H, Wilson C, Corey L: Neonatal herpes simplex virus infection in King County, Washington. Increasing incidence and epidemiologic correlates. JAMA 250:3059–3062, 1983.
3. Prober C, Hensleigh P, Boucher F, Yasukawa L, Au D, Arvin A: Use of routine viral cultures at delivery to identify neonates exposed to herpes simplex virus. N Engl J Med 318:887–891, 1988.
4. Whitley R, Corey L, Arvin A, et al.: Changing presentation of herpes simplex virus infection in neonates. J Infect Dis 158:109–116, 1988.
5. Whitley R, Arvin A, Prober C, et al.: Predictors of morbidity and mortality in neonates with herpes simplex virus infections. N Engl J Med 324:450–454, 1991.
6. Stone K, Brooks C, Guinan M, Alexander E: National surveillance for neonatal herpes simplex virus infections. Sex Transm Dis 16:152–156, 1989.
7. American College of Obstetricians and Gynecologists, Washington, DC: Perinatal herpes simplex virus infections. Technical Bulletin No. 122, November 1988.
8. Reeves W, Corey L, Adams H, Vontver L, Holmes K: Risk of recurrence after first episodes of genital herpes. N Engl J Med 305:315–319, 1981.
9. Corey L, Adams H, Brown Z, Holmes K: Genital herpes simplex virus infections: Clinical manifestations, course and complications. Ann Intern Med 98:958–972, 1983.
10. Lafferty W, Coombs R, Benedetti J, Critchlow C, Corey L: Recurrences after oral and genital herpes simplex virus infection. Influence of site of infection and viral type. N Engl J Med 316:1444–1449, 1987.
11. Johnson R, Nahmias A, Magder L, Lee F, Brooks C, Snowden C: A seroepidemiologic survey of the prevalence of herpes simplex virus type 2 infection in the United States. N Engl J Med 321:7–12, 1989.
12. Kulhanjian J, Soroush V, Au D, et al.: Identification of women at unsuspected risk of primary infection with herpes simplex virus type 2 during pregnancy. N Engl J Med 326:916–920, 1992.
13. Sullender W, Yasukawa L, Schwartz M, et al.: Type-specific antibodies to herpes simplex virus type 2 (HSV-2) glycoprotein G in pregnant women, infants exposed to maternal HSV-2 infection at delivery, and infants with neonatal herpes. J Infect Dis 157:164–171, 1987.
14. Boucher F, Yasukawa L, Bronzan R, Hensleigh P, Arvin A, Prober C: A prospective evaluation of primary genital herpes simplex virus type 2 infections acquired during pregnancy. Pediatr Infect Dis J 9:499–504, 1990.
15. Brown Z, Vontver L, Benedetti J, et al.: Effects on infants of a first episode of genital herpes during pregnancy. N Engl J Med 317:1246–1251, 1987.
16. Trofatter K Jr, Daniels C, Williams R Jr, Gall S: Growth of type 2 herpes simplex in newborn and adult mononuclear leukocytes. Intervirology 11:117–123, 1979.
17. American College of Obstetricians and Gynecologists, Washington, DC: Gynecologic herpes simplex virus infections. Technical Bulletin No. 119, August 1988.
18. Mudido P, Marshall G, Howell R, Schmid D, Steger S, Adams G: Disseminated herpes simplex virus infection during pregnancy. J Reprod Med 38:964–968, 1993.
19. Vontver L, Hickok D, Brown Z, Reid L, Corey L: Recurrent genital herpes simplex virus infection in pregnancy: Infant outcome and frequency of asymptomatic recurrences. Am J Obstet Gynecol 143:75–84, 1982.
20. Brown Z, Vontver L, Benedetti J, et al.: Genital herpes in pregnancy: Risk factors associated with asymptomatic viral shedding. Am J Obstet Gynecol 153:24–30, 1985.
21. Harger J, Amortegui A, Meyer M, Pazin G: Characteristics of recurrent genital herpes simplex infections in pregnant women. Obstet Gynecol 73:367–372, 1989.
22. Prober C, Sullender W, Yasukawa L, Au D, Yeager A, Arvin A: Low risk of herpes simplex virus infections in neonates exposed to the virus at the time of vaginal delivery to mothers with recurrent genital herpes simplex virus infections. N Engl J Med 316:240–244, 1987.
23. Brown Z, Benedetti J, Ashley R, et al.: Neonatal herpes simplex virus infection in relation to asymptomatic maternal infection at the time of labor. N Engl J Med 324: 1247–1252, 1991.
24. Yeager A: Genital herpes simplex infections: Effect of asymptomatic shedding and latency on management of infections in pregnant women and neonates. J Invest Dermatol 83:53–56, 1984.
25. Wittek A, Yeager A, Au D, Hensleigh P: Asymptomatic shedding of herpes simplex virus from the cervix and lesion site during pregnancy. Correlation of antepartum shedding with shedding at delivery. Am J Dis Child 138:439–442, 1984.
26. Arvin A, Hensleigh P, Prober C, et al.: Failure of antepartum maternal cultures to predict the infant's risk of exposure to herpes simplex virus at delivery. N Engl J Med 315:796–800, 1986.
27. Moseley R, Corey L, Benjamin D, Winter C, Remington M: Comparison of viral isolation, direct immunofluorescence, and indirect immunoperoxidase techniques for detection of genital herpes simplex virus infection. J Clin Microbiol 13:913–918, 1981.
28. Hardy D, Arvin A, Yasukawa L, et al.: Use of polymerase chain reaction for successful identification of asymptomatic genital infection with herpes simplex virus in pregnant women at delivery. J Infect Dis 162:1031–1035, 1990.
29. Gibbs R, Mead P: Preventing neonatal herpes—Current strategies. N Engl J Med 326:946–947, 1992.
30. Hutto C, Arvin A, Jacobs R, et al.: Intrauterine herpes simplex virus infections. J Pediatr 110:97–101, 1987.
31. Baldwin S, Whitley R: Teratogen update: Intrauterine herpes simplex virus infection. Teratology 39:1–10, 1989.
32. Corey L, Stone E, Whitley R, Mohan K: Difference between herpes simplex virus type 1 and type 2 neonatal encephalitis in neurologic outcome. Lancet 1:1–4, 1988.

33. Bradley J, Yeager A, Dyson D, Hensleigh P, Medearis A: Neutralization of herpes simplex virus by antibody in amniotic fluid. Obstet Gynecol 60:318–321, 1982.
34. Randolph A, Washington E, Prober C: Cesarean delivery for women presenting with genital herpes lesions. Efficacy, risks and costs. JAMA 270:77–82, 1993.
35. Whitley R: Herpes simplex virus infections. In: Remington J, Klein J (eds): Infectious Diseases of the Fetus and Newborn Infant. 3rd ed. Philadelphia: W. B. Saunders Co., pp 282–305, 1990.
36. Binkin N, Koplan J, Cates W Jr: Preventing neonatal herpes. The value of weekly viral cultures in pregnant women with recurrent genital herpes. JAMA 251:2816–2821, 1984.
37. Gibbs R, Amstey M, Sweet R, Mead P, Sever J: Management of genital herpes infection in pregnancy. Obstet Gynecol 71:779–780, 1988.
38. Andrews E, Yankaskas B, Cordero J, Schoeffler K, Hampp S: Acyclovir in pregnancy registry: Six years' experience. Obstet Gynecol 79:7–13, 1992.
39. U.S. Department of Health and Human Services: Pregnancy outcomes following systemic prenatal acyclovir exposure—June 1, 1984–June 30, 1993. MMWR 42:806–809, 1993.
40. Stray-Pedersen B: Acyclovir in late pregnancy to prevent neonatal herpes simplex. Lancet 336:756, 1990.
41. Scott L, Jackson G, Sanchez P, Custaneda Y, Hall M, Wendel G: Prevention of cesarean section for recurrent genital herpes simplex virus (HSV) using acyclovir suppressive therapy. Abstract No. S223. Society for Gynecologic Investigation, 1993, Toronto, ON.
42. Haddad J, Langer B, Astruc D, Messer J, Lokiec F: Oral acyclovir and recurrent genital herpes during late pregnancy. Obstet Gynecol 82:102–104, 1993.
43. Bernstein D, Lovett M, Bryson Y: The effects of acyclovir on antibody response to herpes simplex virus in primary genital infections. J Infect Dis 150:7–13, 1984.
44. Utley K, Bromberger P, Wagner L, Schneider H: Management of primary herpes in pregnancy complicated by ruptured membranes and extreme prematurity: Case report. Obstet Gynecol 69:471–473, 1987.
45. Major C, Towers C, Lewis D, Asrat T: Expectant management of patients with both preterm premature rupture of membranes and genital herpes. Abstract No. 16. Society of Perinatal Obstetricians. Am J Obstet Gynecol 164: 248, 1991.
46. Nahmias A, Josey W, Naib Z, Freeman M, Fernandez R, Wheeler J: Perinatal risk associated with maternal genital herpes virus infection. Am J Obstet Gynecol 110: 825–837, 1971.
47. Amstey M, Monif G: Genital herpes virus infection in pregnancy. Obstet Gynecol 44:394–397, 1974.
